# A programme of support for care assistants of children admitted with cerebral palsy

**DOI:** 10.4102/ajod.v13i0.1461

**Published:** 2024-12-13

**Authors:** Lebogang L. Molefe, Leepile A. Sehularo, Magdalena P. Koen

**Affiliations:** 1 Department of Nursing, Faculty of Health Sciences, Sefako Makgatho University, Pretoria, South Africa; 2 Department of Nursing, Faculty of Health Sciences, North-West University, Mafikeng, South Africa

**Keywords:** care assistant, cerebral palsy, children, programme, support

## Abstract

**Background:**

Cerebral palsy affects children’s movement and posture because of damage to the brain’s development. Care assistants in healthcare facilities provide care to children. Caring for the children is overwhelming, hence support is required. Such support is absent, causing frustration among care assistants, which leads to poor quality care for children.

**Objectives:**

To explore and describe the experiences of care assistants of children admitted with cerebral palsy in healthcare facilities of the Gauteng province, and to develop a support programme for care assistants.

**Method:**

A qualitative, exploratory, descriptive, and contextual research design was used. Participants were selected from healthcare facilities in Gauteng province. Semi-structured interviews were used to collect data. Content data analysis was used to analyse data. The results were used to develop a support programme for care assistants.

**Results:**

Three themes emerged, namely, a lack of training opportunities, a lack of resources, and a lack of support. The results were used to develop a support programme, using the three steps of the Donabedian model for care: structure, process and outcome.

**Conclusion:**

Care assistants are not given training opportunities, work with limited resources and are not supported, hence the development of a support programme. If effectively utilised, the programme can lead to staff satisfaction and improvement of quality care for children.

**Contribution:**

The study enabled managers in healthcare facilities to see the need for policy and the need for support strategies for care assistants. A support programme was further developed.

## Introduction

Cerebral palsy refers to a group of permanent disorders of the development of movement and posture, causing activity limitations that are attributed to non-progressive disturbances or damage that occurred in the developing foetal or infant brain (Sadowska et al. 2020). Persistence of primitive reflexes or primary patterns that prevent or delay the typical progression of motor development beyond the expected age is a key clinical characteristic of cerebral palsy, manifesting itself with the neurological impairment of the motor system of a child, with characteristics such as spasticity, dyskinesia, hypotonia, ataxia and sometimes dysphagia (Patel et al. 2020). However, not all children with cerebral palsy share the same profile because others may be mobile, play sports, attend university and even marry. However, others may never roll, feed or speak, and remain dependent on others for all activities of daily living. Therefore, they are classified as having predominantly disorders of movement that further include a spectrum of abnormalities such as poor balance and sensory deficits. This study thus focusses on the latter. Thus, children cannot perform activities of daily living that children their age can do. They cannot feed or bathe themselves, walk, talk, button clothes or pick up utensils; hence, they rely on other people for the fulfilment of activities of daily living.

The global prevalence of cerebral palsy estimates ranges from 1 to 4 per 1000 live births. Approximately 18 million people of all ages have cerebral palsy globally, and the prevalence in African countries is suggested to be higher than in Western countries (Malek, Rosenbaum & Gorter 2020). In South Africa, the prevalence of cerebral palsy has been estimated to be as high as 10 per 1000 children (Katangwe et al. 2020), with Gauteng province having the highest number compared to other provinces (Statistics South Africa 2022). The population in Gauteng province is 15.10 million. The province has the highest number of children with intellectual disabilities, including cerebral palsy (Stats SA 2022). According to Stats SA (2022), percentages of children with intellectual disabilities by province are Gauteng 8.3%, KwaZulu-Natal 6.2%, Eastern Cape 4.2%, Western Cape 3.7%, Limpopo 3.4%, Mpumalanga 2.6%, North West 2.3%, Free State 1.7% and Northern Cape 0.7%. The children are admitted to the hospitals for the provision of care because caring at home is difficult for parents and guardians (Guimarães et al. 2023; Sankombo 2023; Smith & Blamires 2022).

When admitted to hospitals, children are cared for by care assistants, who perform basic tasks such as bathing, feeding, changing nappies, changing positions, playing with children and providing stimulation. The Care Business Associate Training (CBAT) addressed the global context of care assistants’ qualification requirements. According to the organisation, there is no legal requirement for care staff to have any particular General Certificate of Secondary Education (GCSE), A-levels or degrees to work within the role in the care sector. However, they must have completed appropriate training to equip them with a standard level of knowledge, skill and experience before they begin working within the sector (CBAT 2023). Similarly, in South Africa, the training and registration of categories of nurses are controlled by the South African Nursing Council (SANC), and this does not include the training of care assistants. There are no formal qualifications required to become a care assistant; however, one needs to have a caring nature and good communication skills. Managers need to send care assistants for training in caring and communication to promote competency in the provision of quality care. Because of the lack of a controlling body, most companies do not adhere to such requirements. Several studies have found that caring for children with cerebral palsy is challenging and demanding. Care assistants experience financial challenges, physical health problems, negative attitudes by other health professionals, stress, emotional trauma and a lack of recognition by employers (Dlamini, Chang & Nguyen 2023; Eloreidi et al. 2021; Manyuma et al. 2023). There is no evidence of support for care assistants.

The theory underpinning the study is social exchange theory which suggests that relationships are based on cost-benefit analysis, meaning each person seeks to maximise their benefits and is expected to reciprocate for the benefits they should have received. When risks outweigh potential rewards, relationships may be abandoned (Ahmad et al. 2022). Therefore, if care assistants do not receive support from their employers while providing care to children, they might feel demotivated and stressed and might abandon the commitment to provide quality care to children.

### Objectives of the study

- To explore and describe the experiences of care assistants of children admitted with cerebral palsy in the Gauteng province.
- To develop a programme to support care assistants of children admitted with cerebral palsy.

## Research methods and design

### Design

An exploratory descriptive design was used to explore and describe the experiences of care assistants of children admitted with cerebral palsy in the Gauteng province. The experiences of care assistants have limited coverage in the medical literature. Before the study, little was known about the experiences of care assistants of children with cerebral palsy (Hunter, McCallum & Howes 2019). In sharing the experiences, coupled with the lack of support for care assistants, the researcher deduced the type of support that care assistants require, and this was further confirmed with care assistants, hence the development of a programme of support. A support programme will offer psychosocial support that will reduce high levels of stress, reduce social isolation, and connect caregivers to patients (Joo et al. 2022). A study by Soto-Rubio et al. (2022) further affirms that support programmes are effective in reducing anxiety, emotional distress and burdens of those who provide care. Support programmes increase knowledge and understanding, enhance efficiency and strategies to apply when faced with challenges (Van der Westhuizen et al. 2020).

### Setting

The study was conducted in the Gauteng province, one of the nine provinces of South Africa. The province is the smallest in size, but the highest in population and children with cerebral palsy (Stats SA 2022). The majority of people in the province opt for government hospitals because of unemployment, competition for scarce resources and inability to afford to send their children to private hospitals. This causes overcrowding in government hospitals, which is further aggravated by limited human resources, including care assistants. It is for this reason that the researcher opted to conduct the study in the province’s three metropolitan municipalities, namely Johannesburg, Tshwane and Ekurhuleni. Six healthcare facilities that focus on children with cerebral palsy within the three municipalities were selected. Table 1 provides a summary of the setting of the study, which is the three metropolitan municipalities, and six healthcare facilities within those municipalities.

**TABLE 1:** Setting.

Number	Metropolitan municipality	Healthcare facility
1.	Johannesburg Metropolitan Municipality	1. Takalani Home for Mentally Handicapped 2. Tara Hospital 3. Helen Joseph Hospital
2.	Tshwane Metropolitan Municipality	1. Cullinan Care and Rehabilitation Centre
3.	Ekurhuleni Metropolitan Municipality	1. Little Eden, Elvira Rota Village (ERV) Bapsfontein 2. Little Eden, Domitilla and Danny Hyams Home (DDHH) Edenvale

*Source:* Molefe, L.L., Sehularo, L.A. & Koen, D.M., 2024, ‘Perspectives of practitioners on support for caregivers of children with intellectual disability’, *Curationis* 47(1), e1–e11. https://doi.org/10.4102/curationis.v47i1.2559

### Study population and sampling strategy

A non-probability purposive sampling technique was used. This technique is used when participants are selected because they have characteristics that the researcher needs in their sample. This means that not all members of a population have an equal chance of participating in the study (Ames, Glenton & Lewis 2019). A target population was care assistants who provided care to children with cerebral palsy. For easy access, the researchers applied a setting to include only care assistants who provide care to children with cerebral palsy in the three metropolitan municipalities of Gauteng province because this is where the gap of a lack of support for care assistants was identified. The principal researcher approached the chief executive officers of facilities and requested permission to use facilities as research areas. The researcher managed to access the wards where care assistants were allocated and requested voluntary participation in the study after a thorough explanation of the purpose, risks and benefits of the study. Fifty care assistants who meet the inclusion criteria were approached and requested to participate. Care assistants were the providers of care to children with cerebral palsy in the six facilities, and had experience of 2 years and above in caring. Because of voluntary participation, only 20 agreed to participate, and for those who refused, their right to voluntary participation was respected. A summary of participants by municipality and healthcare facility is provided in Table 2.

**TABLE 2:** Participants by municipality and healthcare facility.

Metropolitan municipality	Healthcare facility	Number of participants			
		Requested to participate	Who agreed to participate	Female	Male
Johannesburg Metropolitan Municipality	1. Takalani Home for Mentally Handicapped	11	4	2	2
	2. Tara Hospital	6	2	1	1
	3. Helen Joseph Hospital	5	1	1	0
	Total participants in Johannesburg Metropolitan Municipality = **7**				
Tshwane Metropolitan Municipality	1. Cullinan Care and Rehabilitation Centre	15	7	6	1
	Total participants in Tshwane Metropolitan Municipality = **7**				
Ekurhuleni Metropolitan Municipality	1. Little Eden, Elvira Rota Village (ERV) Bapsfontein	7	3	3	0
	2. Little Eden, Domitilla and Danny Hyams Home (DDHH) Edenvale	6	3	3	0
	Total participants in Ekurhuleni Metropolitan Municipality = 6				

*Source:* Molefe, L.L., Sehularo, L.A. & Koen, D.M., 2024, ‘Perspectives of practitioners on support for caregivers of children with intellectual disability’, *Curationis* 47(1), e1–e11. https://doi.org/10.4102/curationis.v47i1.2559

Note: All care assistants who agreed to participate were made to sign the consent forms.

### Data collection

The study is qualitative; therefore, semi-structured individual interviews were conducted to collect data from the 20 participants. Interviews were conducted by the principal researcher, in ward managers’ offices, which were quiet and away from the cubicles of the wards, between September and November 2021, after the ethical clearance certificate was obtained on 17 May 2021. Before the interview, the researcher built rapport by greeting participants with a smile, introducing herself, reassuring the participants that the information shared would be kept confidential, and arranging the chairs so that the researcher and the participant could face each other. Participants were asked if they were still willing to proceed with an interview and were reassured that the interview would not take long, and should the participant experience any discomfort during the session, they should inform the researcher so that the interview could be ended immediately. Each participant was interviewed individually. The audio recorder was used to record the interviews, and consent to record such was obtained from the participants. Participants were reassured that information shared will never be shared with anyone without their consent. The audio recorder and all transcripts were locked in a safe immediately after data collection by the researcher to prevent unauthorised access and were used again by the researcher during data analysis, which occurred immediately after all participants were interviewed.

Each interview lasted between 20 min and 45 min, and no interview exceeded 45 min. The following questions were asked unambiguously and in a neutral tone: ‘How did you experience caring for children with cerebral palsy in the ward? How can you be supported during caring for children with cerebral palsy?’ Participants were encouraged to report their experiences and suggest the support strategies that will work for them. The researcher listened with minimal interruptions and ensured that the answers were to the point. The researcher further noted non-verbal cues and took field notes. All 20 participants were interviewed; however, most participants provided similar responses, indicating data saturation.

### Data analysis

Content analysis was used to analyse data. Six steps of data analysis were followed (Creswell & Creswell 2018). Firstly, the researcher listened to the tape and transcribed the data. A separate file was created for each participant. Every interview was documented separately. Behaviours expressed in participants’ words, facial expressions, gestures and reactions were captured. Notes were recorded immediately after the interview by the researcher, in the participant’s presence, and are duly reflected in the researcher’s analytical memos. Secondly, the researcher organised, ordered and stored data (Molefe 2024). Details of time, location and attendant comments were recorded on all transcripts and field notes. Data were recorded, re-checked and labelled by the researcher. Data were read, and themes, emotions and surprises were considered. Reflective and in-depth reading of the data was done to find supportive evidence for themes. Data was reread to identify elements that might have been overlooked. The researcher then searched for possible alternative meanings and attempted to link discrepancies (Molefe 2024). Thirdly, the researcher coded and categorised data. Coding was used to explore the data and single out words used by participants. The researcher resorted to open coding, which entailed labelling specific pieces of data. Coding was done paragraph by paragraph. The researcher started with a mass of codes that were reduced until each one represented a specific concept. The researcher made use of data reduction to reduce the volume and thereby reduce the list of themes (Molefe 2024). Lastly, themes and sub-themes were identified, presented cohesively and interpreted to produce findings. The researcher used a co-coder who coded independently, following the same steps of data analysis. An online meeting was held between the researcher and the co-coder to discuss identified codes. There were similarities in the themes and sub-themes of the researcher and the co-coder, except in one code where there were differences. Following thorough deliberations, a consensus was reached regarding the acceptable code.

### Trustworthiness

Five testing criteria for trustworthiness were observed: credibility, dependability, confirmability, transferability and authenticity (Molefe, Sehularo & Koen 2022; Polit & Beck 2021). Credibility was attained by transcribing interviews verbatim, involving a co-coder during data analysis, and doing triangulation, achieved by using semi-structured individual interviews, as well as taking field notes. Dependability was attained through the description and application of the research methodology, providing an audit trail and involving a co-coder during data analysis to verify the findings. Confirmability was attained by ensuring that the researcher’s bias, motivation or interest did not shape the findings; hence, the researchers ensured that the data represented the information that participants provided. Transferability was attained through a thick description of the research methodology and triangulation. The study will be published with sufficient data to enable readers to conclude whether transferability can be possible. Authenticity was attained by ensuring that the report conveys perceptions of care assistants and nothing more (Molefe, Sehularo & Koen 2024).

### Ethical considerations

Ethical clearance to conduct this study was obtained from the North West University Health Research Ethics Committee (NWU-HREC) of (No. NWU-00462-20-A1). Permission to access the facilities was granted by the chief executive officers of the six facilities. The ward managers permitted interviews with care assistants and even provided their offices for the discussions. Participants gave their voluntary informed consent to participate and agreed to be audio-recorded. Participants’ names were not used; instead, codes were assigned, for example, P-1.

## Results

Of the 20 who agreed to participate, 4 were males and 16 were females. Table 3 provides a summary of the identifying characteristics of the participants.

**TABLE 3:** Demographic information of participants.

Participant	Age (years)	Gender	Employment title	Employment municipality	Years of experience of caring
P-1	52	Female	Care Assistant	Johannesburg metro	10
P-2	34	Male	Care Assistant	Johannesburg metro	7
P-3	54	Female	Care Assistant	Johannesburg metro	8
P-4	51	Female	Care Assistant	Johannesburg metro	12
P-5	30	Female	Care Assistant	Tshwane metro	6
P-6	53	Female	Care Assistant	Tshwane metro	14
P-7	33	Female	Care Assistant	Tshwane metro	7
P-8	30	Female	Care Assistant	Tshwane metro	6
P-9	28	Female	Care Assistant	Ekurhuleni metro	5
P-10	31	Female	Care Assistant	Ekurhuleni metro	7
P-11	31	Female	Care Assistant	Ekurhuleni metro	10
P-12	30	Female	Care Assistant	Ekurhuleni metro	8
P-13	36	Female	Care Assistant	Ekurhuleni metro	6
P-14	33	Female	Care Assistant	Ekurhuleni metro	9
P-15	42	Male	Care Assistant	Tshwane metro	5
P-16	29	Female	Care Assistant	Tshwane metro	4
P-17	32	Female	Care Assistant	Tshwane metro	7
P-18	40	Female	Care Assistant	Johannesburg metro	8
P-19	28	Male	Care Assistant	Johannesburg metro	9
P-20	43	Male	Care Assistant	Johannesburg metro	12

*Source:* Molefe, L.L., Sehularo, L.A. & Koen, D.M., 2024, ‘Perspectives of practitioners on support for caregivers of children with intellectual disability’, *Curationis* 47(1), e1–e11. https://doi.org/10.4102/curationis.v47i1.2559

The results revealed three themes: a lack of training opportunities, a lack of resources and a lack of support. Themes had sub-themes identified. Table 4 provides a summary of identified themes and sub-themes.

**TABLE 4:** Themes and sub-themes.

Themes	Sub-themes
1. A lack of training opportunities	1.1 Unavailability of workshops 1.2 Unavailability of in-service-training 1.3 Denied study leave opportunities
2. A lack of resources	2.1 Absence of medical equipment to provide care 2.2 Staff shortage
3. A lack of support	3.1 Absence of counselling services 3.2 No appreciation, incentives or recognition

*Source:* Molefe, L.L., Sehularo, L.A. & Koen, D.M., 2024, ‘Perspectives of practitioners on support for caregivers of children with intellectual disability’, *Curationis* 47(1), e1–e11. https://doi.org/10.4102/curationis.v47i1.2559

### Theme 1: A lack of training opportunities

Training refers to short-term courses designed to give employees additional knowledge, practice skills and improve their work performance (Molefe et al. 2024). Training may further continue beyond initial competence to maintain, upgrade and update skills throughout working life (Himam 2022). The three sub-themes are non-attendance of workshops, non-attendance of in-service training and no study leave opportunities.

#### Sub-theme 1.1: Unavailability of workshops

A workshop is a structured and interactive session designed to create an environment for meaningful work and to guide people through a process that will lead to great outcomes. The aim is to encourage learning, collaboration, problem-solving or the creation of new ideas (Matthews 2022:399). Participants cited that they were not given opportunities to attend workshops:

‘Managers do not want to send us to workshops. They always cite a lack of funds and shortage.’ (P-4, 51 years, female, care assistant with 12 years’ experience)

‘We are clueless when it comes to cerebral palsy because we were not trained in psychiatry, hence we do not provide quality care to children.’ (P-7, 51 years, female, care assistant with 7 years’ experience)

Participants further emphasised the need to attend workshops, and further recommended that managers consider sending them to attend workshops:

‘It is important for us to attend workshops because they will help us to improve our skills and knowledge regarding cerebral palsy.’ (P-11, 31 years, female, care assistant with 10 years’ experience)

‘Managers must consider sending us for workshops that will improve our skills and knowledge.’ (P-18, 40 years, female, care assistant with 8 years’ experience)

#### Sub-theme 1.2: Unavailability of in-service training

In-service training is an essential component of vocational education and training in South Africa. It provides learners with the opportunity to apply theoretical knowledge and practical skills in a real workplace environment (Jackson, Jowsey & Honey 2019). Participants cited that they were not given opportunities to attend in-service training on cerebral palsy:

‘We are not given opportunities to attend in-service training. Only professional nurses are prioritised when there is training.’ (P-1, 52 years, female, care assistant with 10 years’ experience)

‘Only if they can allow us to attend in-service training, we can improve in provision of care to the children.’ (P-13, 36 years, female, care assistant with 6 years’ experience)

Participants further cited that in-service training is necessary for them so that they can improve their skills and knowledge relevant to caring for children with cerebral palsy:

‘I am convinced that information provided during in-service training can assist us to improve knowledge and skill of cerebral palsy.’ (P-20, 43 years, male, care assistant with 12 years’ experience)

‘Managers need to invest in regular in-service training because, for those of us that were not trained to care for children with intellectual disabilities, the training will assist us a lot.’ (P-6, 53 years, female, care assistant with 14 years’ experience)

#### Sub-theme 1.3: Denied study leave opportunities

Study leave means leave days provided to employees to pursue undergraduate or postgraduate studies, or professional training which will increase or broaden the competencies of employees (Kauhanen 2020:653). According to participants, they were denied study leaves, hence their knowledge of cerebral palsy was not broadened:

‘When we apply for study leave, we are being denied.’ (P-2, 34 years, male, care assistant with 7 years’ experience)

‘It is very painful that we lack knowledge of cerebral palsy, but when we ask for study leave to engage in programmes that teach care for children with cerebral palsy, our manager refuses.’ (P-15, 42 years, male, care assistant with 5 years’ experience)

Participants further recommended that managers grant them study leave days to enrol in courses about cerebral palsy. They believe that upon completion, the facility will benefit as they will be able to provide quality care to children:

‘They must grant us study leave so that we improve our skills on how to care for children.’ (P-7, 33 years, female, care assistant with 7 years’ experience)

### Theme 2: A lack of resources

The resource is a non-directional physical, psychological, social or organisational characteristic that functions to achieve work goals or reduce demands on the physiological and psychological costs of work (Lee, Rocco & Shuck 2019:10; Molefe et al. 2024). Participants cited a lack of resources.

#### Sub-theme 2.1: Absence of medical equipment to provide care

Medical equipment includes articles, instruments, apparatus or machines used in the prevention, diagnosis, treatment and management of diseases, or for detecting, measuring, restoring, correcting, or modifying the structure or function of the body for some health purpose (Molefe et al. 2024; Zonani et al. 2021). The absence of medical equipment frustrated the participants:

‘There are no wheelchairs, lifting machines, pressure sores cushions for children.’ (P-15, 42 years, male, care assistant with 5 years’ experience)

‘Our backs are painful due to lifting children because there is no equipment that assists in lifting.’ (P-17, 32 years, female, care assistant with 7 years’ experience)

Recommendations for purchasing equipment were made by participants:

‘Managers need to buy medical equipment that will assist us to render quality care, and not to injure ourselves.’ (P-9, 28 years, female, care assistant with 5 years’ experience)

#### Sub-theme 2.2: Staff shortage

In health facilities, adequate staffing is key. The basic principle is that healthcare providers must have sufficient staff on duty to provide care safely and effectively (Ball & Griffiths 2022:872; Molefe et al. 2024). Participants cited that their facilities have insufficient staff:

‘There is a serious shortage. We are only four, and we take care of fifty children.’ (P-13, 36 years, female, care assistant with 6 years’ experience)

‘One person is allocated to care for close to ten children alone. The children are heavy, and the job is too much for one person to manage.’ (P-18, 40 years, female, care assistant with 8 years’ experience)

‘We cannot even take leave because of shortage.’ (P-3, 54 years, female, care assistant with 8 years’ experience)

Participants pleaded for managers to employ more care assistants:

‘They must employ more staff to ease the burden of caring.’ (P-12, 30 years, female, care assistant with 8 years’ experience)

‘Caring for helpless children is hectic. More care assistants must be employed.’ (P-15, 42 years, male, care assistant with 5 years’ experience)

‘They must request increment of budget so that they employ more staff.’ (P-4, 42 years, female, care assistant with 12 years’ experience)

### Theme 3: A lack of support

Support is showing care and compassion for another person, and it is a critical way of achieving patient-centred care, and a lack of staff support contributes to poor delivery of care for patients (Bradshaw et al. 2022). Participants cited a lack of support, particularly emotional support.

#### Sub-theme 3.1: Absence of counselling services

Counselling is a process that involves a trained counsellor helping individuals to find ways to work through and understand their problems. It promotes a healthy lifestyle and improves the quality of life and overall health (Kariemlou et al. 2019; Molefe et al. 2024). According to participants, the facilities had no counselling services where their emotional well-being could be addressed:

‘We are overwhelmed, stressed, and frustrated. There are no counselling services. We always ask to be referred for counselling, but nobody is listening.’ (P-11, 31 years, female, care assistant with 10 years’ experience)

‘Caring for these children is emotionally draining and depressing. We need de-briefing sessions, but our pleas are being ignored.’ (P-6, 53 years, female, care assistant with 14 years’ experience)

Participants further recommended counselling services in the facility:

‘Managers must organise counselling service of psychologists or professional counsellor.’ (P-18, 40 years, female, care assistant with 8 years’ experience)

‘We must be allowed de-briefing sessions once a week.’ (P-16, 29 years, female, care assistant with 4 years’ experience)

#### Sub-theme 3.2: No appreciation, incentives or recognition

Incentives, recognition and appreciation are things that motivate or encourage a person to do something and can include a payment, or a concession to stimulate greater output or investment. They are the reasons employees feel energetic and motivated towards their work (Liu & Liu 2022; Molefe et al. 2024). Participants cited the lack of the three in their facilities:

‘We do not get incentives for work well done, not even verbal appreciation, yet we work so hard.’ (P-10, 31 years, female, care assistant with 7 years’ experience)

‘Managers always cite lack of funds being the reason we cannot get incentive. This is not fair.’ (P-13, 36 years, female, care assistant with 6 years’ experience)

‘Even a certificate of appreciation will motivate us because we cannot be rewarded with money because we are told that there is not enough budget.’ (P-20, 43 years, male, care assistant with 12 years’ experience)

Participants further recommended the need for managers to introduce incentive bonuses to motivate staff:

‘Managers must request an increase in budget so that they give us incentives at the end of the year. Many companies reward their employees at the end of the year, and this motivates staff to even work harder. For us, there is nothing that motivates us.’ (P-2, 34 years, male, care assistant with 7 years’ experience)

## Discussion

The first objective of this study was to explore and describe the experiences of care assistants of children admitted with cerebral palsy. Identified themes with their sub-themes provided insight into what care assistants experience when caring for children with cerebral palsy. The lack of knowledge as experienced by care assistants, is a revelation regarding the importance of training opportunities in the working environment. Training can be in the form of workshops, in-service training and study leave opportunities. Employee training and development serves as a tool that not only enhances the competencies required to perform a job, but also provides a means to assist individuals in feeling more satisfied with the results of their performance (Rodriguez & Walters 2017). A lack of training therefore contributes to poor job performance and low self-esteem (Yimam 2022). Workshop attendance is important in acquiring state-of-the-art knowledge on external developments as a dominant source of competitive advantage for the organisation. Furthermore, attending workshops is an important way of learning from other professionals in the same occupational field outside the organisation where one is employed (De Grip & Pleijers 2019). In-service training, on the other hand, assists in maintaining, upgrading and updating skills throughout working life (Himam 2022). Managers must therefore allow employees to attend training and workshops to better their performance in caring (Yimam 2022) and further approve study leave applications.

A lack of resources as experienced by care assistants is a trigger for frustration during the provision of care. When the resources to provide quality care are limited or unavailable, healthcare workers become overwhelmed. With healthcare resources, people’s life expectancy increases, and overall mortality declines (Raghupathi & Raghupathi 2023). A shortage of resources such as special wheelchairs, lifting machines, supporting cushions, linen, disposable nappies, special feeding utensils and staff shortage become a barrier that may reduce access to health services and increase the risk of poor health outcomes (Qiu et al. 2022). It is advisable for the managers to review the budget and request more funding so that the issue of inadequate resources can be adequately addressed.

A lack of support experienced by care assistants, especially emotional support, predisposes staff members to psychological difficulties. Without emotional support, the psychological well-being of care assistants may deteriorate, resulting in depression, which can affect the quality of care given to children (Mbugua, Kuria & Ndetei 2021). Managers must implement psychological support strategies such as listening to an employee’s concerns, allowing employees to talk about their emotions and delivering encouragement and guidance to help employees regulate their emotions (Molefe et al. 2024; Pohl et al. 2022). A study by Schlebusch et al. (2024) proves that supporting caregivers of children with developmental disabilities enormously improves the well-being of caregivers. The study used the World Health Organization (WHO) Caregiver Skill Training (CST) Caregiver Well-being module programme to address caregivers’ psychological challenges during caring. The aim was to establish a mindfulness-based behavioural therapy that builds and promotes psychological flexibility. Before the application of the programme, caregivers reported high levels of psychological distress, depression and anxiety. However, after the programme, caregivers showed positive improvements in the expected directions on all the mental health and well-being measures. The programme provides strong evidence that the support initiative improves the well-being of healthcare workers, including care assistants of children with cerebral palsy. The second objective of this study was to develop a support programme for care assistants of children admitted with cerebral palsy, which leads to the next step: the development of the support programme.

### Development of a support programme

A support programme was developed by following the Donabedian model for quality care (2005), considering the aforementioned interview results. Donabedian’s model refers to the environment and the resources necessary to provide services (Lo Porto 2020) and assesses the environment in which healthcare workers perform their duties to ensure care provision. The model is further used to develop and describe a psychosocial model for enhancing psychosocial support for healthcare workers during coronavirus disease 2019 (COVID-19) and other public health emergencies (Moyo, Tshivhase & Mavhundu-Madzusi 2023).

**FIGURE 1:**
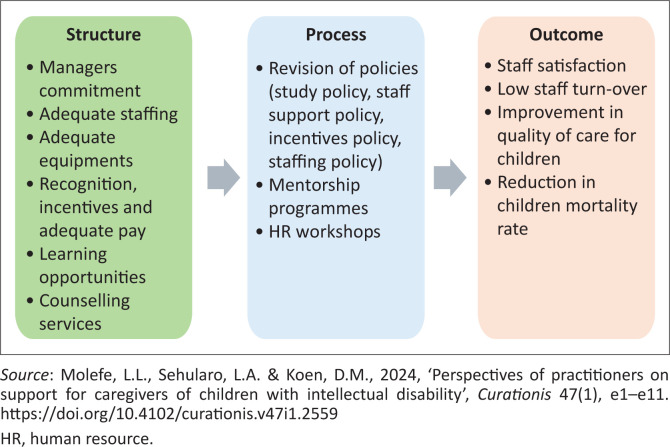
Programme of support for care assistants of children admitted with cerebral palsy in the hospital.

When care providers do not receive the support needed to perform their duty to care, it means a positive outcome on care, which is quality, will never be reached. It is for this reason that the researcher found the model to be relevant for the development of a support programme for care assistants of children admitted with cerebral palsy in the hospital. The model was followed by applying the social exchange theory. Social exchange theory comprises actions contingent on the rewarding reactions of others, which over time provides for mutually rewarding transactions and relationships (Lo Porto 2020). Implications to social change may include improving the organisational policies and procedures that will align with the guidelines specified in the core competencies of the care assistants. Once the core competencies are aligned with organisational policies, job satisfaction, work performance and morale will improve, ultimately enhancing the delivery of healthcare services. Furthermore, this alignment will promote the worth, dignity and development of care assistants. The theory further emphasises that perception plays a crucial role in the implementation of services. Therefore, it is important for care assistants to have a positive perception of their organisation, one that reflects a commitment to human impact, moral goodness and unconditional social improvement (Lo Porto 2020). The model has three steps: structure, process and outcome (SPO). The steps are explained and summarised in Figure 1.

### Structure

The structure includes all factors that affect the care delivery context, such as physical facility, equipment, human resources and organisational characteristics such as staff training and payments (Donabedian 2005; Moyo et al. 2023). Results obtained during data analysis informed the researcher on what structural aspects would impact quality care for children and care assistants. The structure of this study therefore includes managers’ commitment, adequate staffing, adequate equipment, recognitions, incentives and adequate pay, learning opportunities for staff and counselling services for staff. These resources are necessary to not only ensure quality care to the children, but also to ensure the holistic well-being of care assistants, thus supporting care assistants.

### Process

The process is the sum of all actions that make up healthcare, and it contains all acts of bettering healthcare delivery (Donabedian 2005). The process relates to the institutional support that ensures that all healthcare providers experience job satisfaction (Moyo et al. 2023). The identified process of the study includes revision of policies (study policy, staff support policy, incentives policy and staffing policy), mentorship programmes and human resource (HR) workshops. To ensure improvement in quality care for children and the well-being of care assistants, aspects related to the process should be revised to address the identified gaps that hinder quality care for children, which eventually lead to stress and frustration among care assistants.

### Outcome

The outcome is an objective to be achieved (Donabedian 2005). Outcomes are further referred to by Moyo et al. (2023) as the end results that have an effect on the recipients (healthcare workers) in providing care. Evidence of an outcome includes changes to health status, behaviour or knowledge, as well as patient and staff satisfaction, and health-related quality of life. Outcomes of the study include staff satisfaction, low staff turnover, improvement in quality of care for children and reduction in children mortality rate. Achievement of such outcomes will promote the holistic well-being of care assistants.

### Validation of a programme

Before the implementation process, the programme was validated using the Delphi technique. A team of experts, consisting of multidisciplinary members (psychologists, psychiatrists, social workers, professional nurses and researchers) from healthcare facilities, was identified. Validation questionnaires were developed and distributed to the team members. The validation process occurred in three rounds. The first round involved distributing yes or no questions. The second round included structured questions focussing on whether the programme would be beneficial to the care assistants and identifying areas for improvement. In the third round, a summary of the results from the first two rounds was circulated to the participants for review, allowing them to confirm whether the summary reflected their views. All feedback from the experts was positive, confirming that the programme is relevant and will yield positive results.

### Implementation of a programme of support

During implementation of the proposed programme of support, five key areas will be applied, namely, a multidisciplinary team approach, focused management, stakeholders support, open communication, and in-service training. Each key area is designed to address a specific stressor identified by care assistants.

**FIGURE 2:**
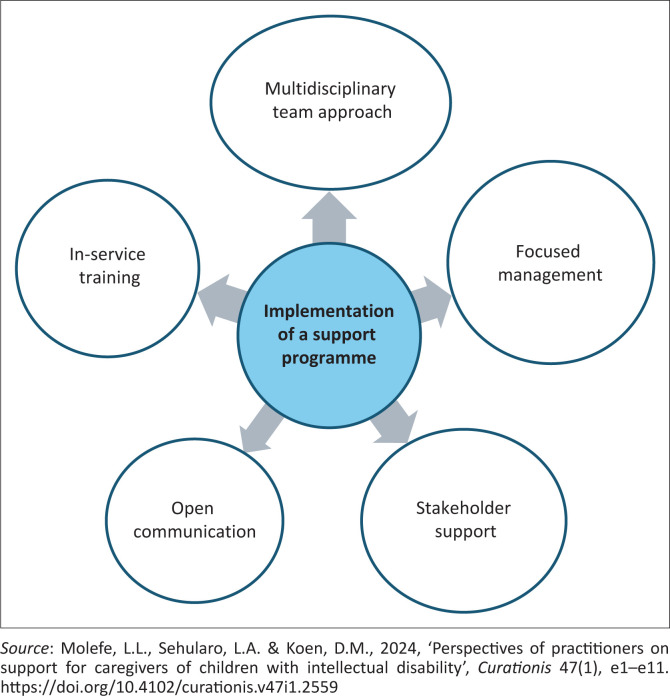
Implementation of a programme of support.

Figure 2 summarises the how, where, by whom and with what resources will the programme be implemented.

The programme advocates that multidisciplinary team members must be committed to supporting care assistants. This includes doctors, physiotherapists, speech therapists, occupational therapists, dietitians, counsellors and any other healthcare professionals involved in the care of children. Each member must be willing to play their part in assisting care assistants in providing care. For example, if a physiotherapist does not visit the children to provide exercises, the responsibility falls on the care assistants. If children develop contractures, care assistants will be the first to be blamed, as they are with the children 24-h a day. This can lead to care assistants feeling overwhelmed and stressed. The second key area focusses on effective management. Managers are expected to support caregivers by being empathetic and listening to the concerns raised by care assistants. They should assist care assistants in negotiating a better remuneration package with the human resources department and review and revise policies that disadvantage care assistants. Stakeholder support means that entities such as the Department of Health must back facilities caring for children by providing an adequate budget to ensure that both human and material resources are sufficient. There must be open communication among everyone directly involved in care. For example, the manager needs to create an environment where care assistants can express their concerns, frustrations and dissatisfactions without fear of retaliation or discrimination. Managers should ensure that care assistants have opportunities to attend in-service training and workshops, which will enhance their self-esteem and confidence in providing care. Mental healthcare professionals in various hospitals in Gauteng were asked to validate the programme. It was found to be clear, feasible and relevant to achieving the goal of supporting care assistants.

### Limitations

The study was confined to one province of the country, leaving the experiences of care assistants in other provinces unknown. Consequently, because of the qualitative nature of the research, the findings cannot be generalised to other provinces. However, the results are likely to apply to other settings in South Africa, as the identified themes address universal issues that can be used to improve the conditions of care assistants nationally.

## Conclusion

The study explored and described the experiences of care assistants working with children admitted with cerebral palsy. Three themes emerged, namely, a lack of training opportunities, a lack of resources and a lack of support. The conclusion is that care assistants for children with cerebral palsy do not have adequate skills and knowledge to provide care because they are not afforded opportunities to improve their skills and knowledge, there are no resources to provide care and they are not supported. These findings were used to develop a support programme for care assistants. If effectively utilised, the support programme can improve the working conditions of care assistants, thus promoting staff satisfaction and eventually provision of quality care for children. The programme can be applied in other healthcare facilities both nationally and internationally because it is possible that challenges encountered by care assistants could be similar across some countries.
