# Corrigendum: A programme of support for care assistants of children admitted with cerebral palsy

**DOI:** 10.4102/ajod.v14i0.1649

**Published:** 2025-01-13

**Authors:** Lebogang L. Molefe, Leepile A. Sehularo, Magdalena P. Koen

**Affiliations:** 1 Department of Nursing, Faculty of Health Sciences, Sefako Makgatho University, Pretoria, South Africa; 2 Lifestyle Diseases Research Focus Area, Faculty of Health Sciences, North-West University, Mahikeng, South Africa; 3 NuMIQ Research Focus Area, Faculty of Health Sciences, North-West University, Potchefstroom, South Africa

In the published article, A programme of support for care assistants of children admitted with cerebral palsy, there was an error in author 2 and 3s affiliation.

For author 2, instead of:

Department of Nursing, Faculty of Health Sciences, North-West University, Mafikeng, South Africa

It should be:

Lifestyle Diseases Research Focus Area, Faculty of Health Sciences, North-West University, Mahikeng, South Africa

For author 3, instead of:

Department of Nursing, Faculty of Health Sciences, North-West University, Mafikeng, South Africa

It should be:

NuMIQ Research Focus Area, Faculty of Health Sciences, North-West University, Potchefstroom, South Africa

The authors apologise for this error. The correction does not change the study’s findings of significance or overall interpretation of the study’s results or the scientific conclusions of the article in any way.

